# Does chemotherapy improve survival outcomes in breast cancer survivors with secondary primary stage I non-small cell lung cancer? A real-world analysis using machine learning models

**DOI:** 10.3389/fonc.2025.1646580

**Published:** 2025-09-12

**Authors:** Bohao Liu, Lutong Yan, Jiaqi Huang, Xingzhuo Zhu, Jinteng Feng, Deqian Qiao, Na Hao, Guangjian Zhang, Shan Gao

**Affiliations:** ^1^ Department of Thoracic Surgery, The First Affiliated Hospital of Xi’an Jiaotong University, Xi’an, China; ^2^ Key Laboratory of Enhanced Recovery After Surgery of Integrated Chinese and Western Medicine, Administration of Traditional Chinese Medicine of Shaanxi Province, The First Affiliated Hospital of Xi’an Jiaotong University, Xi’an, China; ^3^ Departments of Vascular Surgery of Zhongshan Hospital, Fudan University, Shanghai, China; ^4^ Department of Breast Surgery, The First Affiliated Hospital of Xi’an Jiaotong University, Xi’an, China

**Keywords:** breast cancer, lung cancer, second primary cancer, machine learning, chemotherapy

## Abstract

**Background:**

Advances in breast cancer treatment have prolonged survival, leading to an increased incidence of secondary primary lung cancer (SPLC) in survivors. This study aims to investigate the prognosis and treatment strategies for patients with recurrent early-stage lung cancer histories and establish predictive models to guide clinical practice.

**Methods:**

This study analyzed clinical data from 2,775 patients (2008–2024) extracted from the SEER database and 15 patients (2008–2024) from the cancer registry of the First Affiliated Hospital of Xi’an Jiaotong University. The analysis focused on comparing clinical characteristics, prognosis, and chemotherapy benefits between early-stage second primary lung cancer (SPLC) patients with a history of breast cancer and those with primary lung cancer. The average age of patients in the SEER cohort was 69.64 ± 8.89 years(31-90), while the 15 hospital-registered patients had an average age of 67.15 ± 9.12 years(43-77). We employed neural network-based machine learning methods to develop models for predicting treatment decisions. Specifically, the COX-lung and MLP-lung models were developed, with a LOG-lung model used for comparison.

**Results:**

LC patients with a prior breast cancer history had significantly poorer prognosis survival time of 93 months *vs* 129 months. Postoperative chemotherapy improved the prognosis for some patients; however, the population benefiting from chemotherapy exhibited specific clinical characteristics. The COX-lung and MLP-lung models accurately predicted chemotherapy beneficiaries, with the MLP-lung model achieving an AUC of 0.813 and high positive predictive value.

**Conclusion:**

SPLC with prior breast cancer do have a poorer prognosis than lung cancer patients, although postoperative chemotherapy can benefit some individuals, careful selection of patients to receive chemotherapy is still warranted. We developed COX-lung and MLP-lung models which can predict beneficiaries of chemotherapy, providing crucial insights for clinicians in formulating personalized treatment plans. The findings indicate that this patient population is heterogeneous, necessitating more individualized treatment strategies.

## Highlights

- Breast cancer survivors with early-stage SPLC have poorer prognosis than primary lung cancer patients.
- Postoperative chemotherapy benefits some SPLC patients, but selection is crucial.
- We developed the COX-lung and the MLP-lung model, together can provide accurate identification of chemotherapy beneficiaries.

## 1 Introduction

Breast cancer survivors face a significantly elevated risk of developing second primary lung cancer (SPLC), with a standardized incidence ratio of 1.25 compared to the general population (p<0.001) (1, 2). Among female patients with lung cancer as a second primary, breast cancer is the most common first malignancy (35.1%), and lung cancer emerges as the leading cause of death in this group (3). This heightened risk, together with improved survival from breast cancer (4, 5), highlights a growing clinical challenge: the management of early-stage non-small cell lung cancer (NSCLC) as a second primary malignancy.

Regular follow-up in breast cancer survivors enables earlier detection of stage I NSCLC (6). However, this advantage is overshadowed by a significant clinical dilemma: the lack of evidence-based guidelines for managing early-stage secondary primary lung cancer (SPLC) following breast cancer. While surgical resection is standard for *de novo* stage I NSCLC, its direct application to SPLC remains debatable due to the distinct clinical profiles of these patients. Breast cancer survivors often experience therapy-related immunosuppression, comorbidities, or cumulative toxicities, potentially influencing both prognosis and treatment tolerance (7, 8). Despite this, the role of postoperative systemic therapy—particularly adjuvant chemotherapy—in this context is underexplored. Current guidelines offer little direction on whether and how to adapt treatment strategies based on prior cancer history and therapies received. This clinical scenario remains inadequately studied, leaving clinicians with limited references to guide individualized management. Therefore, identifying relevant risk factors and evaluating the potential benefits of systemic therapy are essential to optimize outcomes for this unique patient population.

Consequently, the current study aims to identify risk factors for secondary primary stage I NSCLC in patients with prior non-metastatic breast cancer and evaluate the potential benefits of systemic therapy for this population. Additionally, the study will develop treatment decision models using diverse approaches to inform clinical decisions. External validation will be conducted using data from our center to assess and identify the optimal model.

## 2 Materials and methods

### 2.1 Patients

A retrospective review was conducted on resectable stage I non-small cell lung cancer (NSCLC) patients from the SEER database, utilizing clinical and pathological data extracted from the database. Classification was based on the ICD-O-3/WHO 2008 criteria for “lung and bronchus.” Additionally, an external validation cohort from our institution was included in this study. Data from our center covered patients with a history of non-metastatic breast cancer who later developed stage I lung cancer, between January 2008 and December 2024. To identify patients with a previous history of breast cancer who were diagnosed with secondary NSCLC, the incidence of breast cancer was traced using patient IDs.

The study population was defined by the following inclusion criteria (1): pathological confirmation of non-small cell lung cancer (excluding small cell carcinoma) (2); definitive stage I classification per AJCC 9th edition, based on complete TNM parameters; and (3) a prior history of stage I-III breast cancer. Patients were excluded if critical information was missing, including incomplete treatment records, undefined survival status, unverified age at diagnosis, or insufficient pathological data to confirm stage I NSCLC. For baseline characteristics with partial missing data (e.g., tumor grade, ER/PR/HER2 status, and other biomarkers), these values were categorized as “Unknown” and retained in the analysis to prevent exclusion bias.

The study received ethical approval from the Institutional Review Board at the First Affiliated Hospital of Xi’an Jiaotong University (Ethical Approval Number: XJTU1AF2024LSYY-112), with exemption from patient informed consent granted due to the retrospective nature of the study. The research adheres to the ethical principles outlined in the Declaration of Helsinki by the World Medical Association.

Baseline demographic information and clinical characteristics (including age, sex, and ethnicity at diagnosis), attributes of the two primary tumors (such as histological type, tumor size, location, and grade), and treatment modalities for both tumors were collected from the databases and cancer registry at our institution. Lung cancer histology was classified into three categories: adenocarcinoma, squamous cell carcinoma, and other types (e.g., large cell carcinoma). The interval was defined as the period between the diagnosis of breast cancer and the subsequent development of early-stage lung cancer. Overall survival (OS) was defined as the time from the diagnosis of primary lung cancer to death from any cause or the date of the last follow-up.

### 2.2 Statistical analysis

The Kaplan-Meier method was employed for prognostic estimation. Logistic regression analysis was conducted to identify risk factors associated with the incidence. Inverse Probability of Treatment Weighting (IPTW) adjusted for bias, and the COX proportional hazards model assessed clinical variables influencing long-term survival. Hazard ratios (HRs) with 95% confidence intervals (CIs) were presented. Continuous variables were presented as mean ± standard deviation (SD) and analyzed with t-tests., while categorical variables with normal distribution were reported as counts and percentages and were examined with Pearson’s chi-square test. Computations and graphical outputs were performed using R (version R-4.2.1) and Python (version Python-3.4). X-title (version 3.6.1) was used to determine potential cut-off points, and Origin (version 2021) generated confusion matrices and ROC curves.

### 2.3 Modeling construction

This study developed one predictive model and three therapeutic decision-making models. The predictive model was based on the critical risk factors identified via the COX proportional hazards model and visualized through a nomogram. Its efficacy assessed by the consistency index (C-index) and calibration plots. Regarding the therapeutic decision-making models: the COX-lung model was developed using the scoring system from the nomogram to distinguish between high-risk and low-risk groups based on cumulative scores. The LOG-lung model was constructed using the logistic regression algorithm, via Python’s “*Logistic regression*” function. The MLP-lung model is a machine learning construct that employs a multi-layer perceptron (MLP) neural network to predict high-risk groups and chemotherapy suitability. The architecture of the MLP neural network as described in this article is depicted in **Supplementary Figure S1**. The structure of the sub-model comprises three layers of nodes (1): input layer with 19 variables (2), hidden layer, and (3) output layer. The number of neurons in the hidden layer ‘n’ is fixed at 50. The model was trained for 500 epochs, with a learning rate η of 0.02. The cross-entropy loss function was employed as the loss metric, while the Adam optimizer was utilized for optimization. To assess the impact of each feature on the model’s output, a gradient based approach was implemented, with the training dataset used for model training. This involved setting requires grad to True for the test data to facilitate gradient computation. The model’s outputs were determined, and the gradients were calculated using the backward method. The meaning of the absolute gradients for each feature was computed to serve as an indicator of feature significance. The three therapeutic decision-making models were all validated against our validation dataset. A confusion matrix was constructed for the COX-lung model, while ROC curves and confusion matrices were generated for the LOG-lung and MLP-lung models. The models’ discriminatory capabilities were quantified using the area under the curve (AUC) derived from the receiver operating characteristic (ROC) analysis.

## 3 Results

### 3.1 Characteristics and risk factors

This study included 2775 secondary primary stage I NSCLC patients with prior non-metastatic breast cancer from the SEER database for survival analysis, and the clinical and pathological features of lung cancer are shown in **Supplementary Table S1**.

Of the 2775 patients, 2686 who underwent surgical treatment were selected and categorized into two groups based on adjuvant chemotherapy administration: Surgery + Chemotherapy (n=388) and Surgery (n=2298). There were significant differences between the two groups in age, interval to tumor diagnosis, histology of lung cancer, lung cancer grade, lung cancer T stage, histological type of breast cancer, HER2 expression status, and history of breast cancer chemotherapy. To address the impact of confounding factors, this study utilized IPTW for correction, with the adjusted data presented in **Table 1**. Moreover, the study also gathered data on 15 secondary primary stage I NSCLC patients with prior non-metastatic breast cancer and had received adjuvant chemotherapy at our hospital, with their clinical and pathological features detailed in **Supplementary Table S2**.

**Table 1:** Baseline characteristics.

Characteristics	Unadjusted sample				IPTW adjusted			
	Surgery+Chemotherapy n =388(%)	Only surgery n =2298(%)	P	SMD	Surgery+Chemotherapy n =2716.07(%)	Only surgery n =2685.70(%)	P	SMD
Age at diagnosis								
≤70	261 (67.27)	1101 (47.91)	<0.001	0.399	1249.43 (46.00)	1359.25 (50.61)	0.158	0.092
>70	127 (32.73)	1197 (52.09)			1466.64 (54.00)	1326.45 (49.39)		
Intervals								
≤24	198 (51.03)	716 (31.16)	<0.001	0.412	887.65 (32.68)	913.75 (34.02)	0.634	0.028
>24	190 (48.97)	1582 (68.84)			1828.42 (67.32)	1771.94 (65.98)		
Histology of LC								
Adenocarcinoma	277 (71.39)	1486 (64.66)	0.01	0.175	1795.78 (66.12)	1765.10 (65.72)	0.966	0.018
Squamous cell carcinoma	65 (16.75)	404 (17.58)			454.70 (16.74)	467.85 (17.42)		
Others	46 (11.86)	408 (17.75)			465.60 (17.14)	452.74 (16.86)		
Grade of LC								
Grade I	61 (15.72)	524 (22.80)	<0.001	0.272	565.56 (20.82)	585.53 (21.80)	0.96	0.046
Grade II	156 (40.21)	1043 (45.39)			1194.02 (43.96)	1195.58 (44.52)		
Grade III	108 (27.84)	446 (19.41)			575.58 (21.19)	553.86 (20.62)		
Grade IV	2 (0.52)	10 (0.44)			8.19 (0.30)	11.81 (0.44)		
Unknown	61 (15.72)	275 (11.97)			372.72 (13.72)	338.92 (12.62)		
Laterality								
Left - origin of primary	159 (40.98)	960 (41.78)	0.811	0.016	1099.69 (40.49)	1117.68 (41.62)	0.736	0.023
Right - origin of primary	229 (59.02)	1338 (58.22)			1616.38 (59.51)	1568.01 (58.38)		
Primary site								
Upper lobe	238 (61.34)	1377 (59.92)	0.776	0.056	1709.92 (62.96)	1616.63 (60.19)	0.814	0.06
Middle lobe	20 (5.15)	117 (5.09)			139.99 (5.15)	137.36 (5.11)		
Lower lobe	123 (31.70)	774 (33.68)			833.96 (30.70)	895.49 (33.34)		
Others	7 (1.80)	30 (1.31)			32.20 (1.19)	36.21 (1.35)		
T stage of LC								
T1a	35 (9.02)	322 (14.01)	<0.001	0.27	276.80 (10.19)	356.11 (13.26)	0.442	0.11
T1b	138 (35.57)	900 (39.16)			1109.58 (40.85)	1037.16 (38.62)		
T1c	68 (17.53)	471 (20.50)			505.92 (18.63)	538.27 (20.04)		
T2a	147 (37.89)	605 (26.33)			823.78 (30.33)	754.16 (28.08)		
Stage of BC								
I	236 (60.82)	1385 (60.27)	0.695	0.048	1656.75 (61.00)	1621.17 (60.36)	0.901	0.03
II	130 (33.51)	756 (32.90)			898.29 (33.07)	886.02 (32.99)		
III	22 (5.67)	157 (6.83)			161.03 (5.93)	178.51 (6.65)		
Histology of BC								
IDC/NST	23 (5.93)	121 (5.27)	0.679	0.029	139.27 (5.13)	143.35 (5.34)	0.875	0.009
Good Prognosis Tissue	365 (94.07)	2177 (94.73)			2576.81 (94.87)	2542.35 (94.66)		
ER								
Positive	300 (77.32)	1839 (80.03)	0.372	0.075	2169.63 (79.88)	2137.49 (79.59)	0.928	0.024
Negative	61 (15.72)	334 (14.53)			383.05 (14.10)	396.64 (14.77)		
Borderline/Unknown	27 (6.96)	125 (5.44)			163.40 (6.02)	151.57 (5.64)		
PR								
Positive	241 (62.11)	1515 (65.93)	0.133	0.106	1737.34 (63.97)	1753.27 (65.28)	0.902	0.028
Negative	111 (28.61)	629 (27.37)			784.86 (28.90)	744.41 (27.72)		
Borderline/Unknown	36 (9.28)	154 (6.70)			193.87 (7.14)	188.02 (7.00)		
HER2								
Positive	24 (6.19)	104 (4.53)	0.02	0.156	131.40 (4.84)	129.34 (4.82)	0.749	0.048
Negative	98 (25.26)	732 (31.85)			899.98 (33.14)	830.51 (30.92)		
Borderline/Unknown	266 (68.56)	1462 (63.62)			1684.69 (62.03)	1725.85 (64.26)		
Grade of BC								
Grade I	87 (22.42)	550 (23.93)	0.42	0.108	691.74 (25.47)	637.48 (23.74)	0.979	0.044
Grade II	146 (37.63)	893 (38.86)			1032.87 (38.03)	1036.73 (38.60)		
Grade III	104 (26.80)	518 (22.54)			608.12 (22.39)	622.86 (23.19)		
Grade IV	2 (0.52)	20 (0.87)			25.78 (0.95)	22.11 (0.82)		
Unknown	49 (12.63)	317 (13.79)			357.56 (13.16)	366.52 (13.65)		
Chemotherapy of BC								
Yes	151 (38.92)	758 (32.99)	0.026	0.124	837.89 (30.85)	906.95 (33.77)	0.325	0.062
No/Unknown	237 (61.08)	1540 (67.01)			1878.18 (69.15)	1778.75 (66.23)		
Radiation of BC								
Yes	209 (53.87)	1203 (52.35)	0.618	0.03	1470.04 (54.12)	1412.34 (52.59)	0.647	0.031
No/Unknown	179 (46.13)	1095 (47.65)			1246.03 (45.88)	1273.36 (47.41)		
Surgery of BC								
Yes	377 (97.16)	2253 (98.04)	0.354	0.057	2656.67 (97.81)	2629.64 (97.91)	0.906	0.007
No/Unknown	11 (2.84)	45 (1.96)			59.41 (2.19)	56.05 (2.09)		

### 3.2 Univariate and multivariate COX

The univariate COX regression analysis revealed that age > 70 years, interval >24 months, non-adenocarcinoma histology, advanced lung cancer T-stage, high lung cancer grade, previous breast cancer grade, and the absence of breast cancer-related radiotherapy and chemotherapy were predictors of poor prognosis. Patients who received chemotherapy in addition to surgery exhibited a more favorable prognosis compared to those who underwent surgery alone. The multivariate analysis further validated that age, T-stage of lung cancer, lung cancer grade, pathological grade of lung cancer, previous breast cancer grade, and chemotherapy were correlated with prognosis (p < 0.05). Notably, the hormone receptor status (PR, ER, HER2) of previous breast cancer did not have a significant impact on the prognosis of lung cancer (p > 0.05). The results of univariate and multivariate COX regression analyses for prognostic factors are presented in **Table 2**.

**Table 2:** Multivariable COX proportional hazard model for survival.

Variables	OS		OS	
	Univariate analysis		Multivariate analysis	
	HR (95% Cl)	P	HR (95% Cl)	P
Age at diagnosis				
≤70	Reference		Reference	
>70	1.743 (1.547-1.962)	<0.001	1.568 (1.381-1.780)	<0.001
Intervals				
≤24	Reference		Reference	
>24	1.214 (1.074-1.373)	0.002	1.103 (0.966-1.260)	0.148
Histology of LC				
Adenocarcinoma	Reference		Reference	
Squamous cell carcinoma	1.827 (1.587-2.102)	<0.001	1.511 (1.297-1.76)	<0.001
Others	0.934 (0.764-1.141)	0.203	0.943 (0.769-1.157)	0.576
Grade of LC				
Grade I	Reference		Reference	
Grade II	1.403 (1.187-1.660)	<0.001	1.262 (1.062-1.500)	0.008
Grade III	1.985 (1.657-2.379)	<0.001	1.696 (1.394-2.064)	<0.001
Grade IV	1.480 (0.696-3.148)	0.308	1.707 (0.793-3.673)	0.217
Unknown	1.389 (1.108-1.741)	0.004	1.357 (1.079-1.706)	0.009
Laterality				
Left - origin of primary	Reference		Reference	
Right - origin of primary	1.009 (0.896-1.136)	0.885	0.996 (0.881-1.125)	0.944
Primary site				
Upper lobe	Reference		Reference	
Middle lobe	1.051 (0.810-1.363)	0.710	1.066 (0.814-1.395)	0.644
Lower lobe	1.031 (0.907-1.171)	0.640	1.027 (0.902-1.168)	0.691
Others	1.025 (0.624-1.683)	0.923	0.974 (0.591-1.604)	0.917
T stage of LC				
T1a	Reference			
T1b	1.351 (1.083-1.684)	0.008	1.306 (1.046-1.631)	0.019
T1c	1.541 (1.217-1.950)	<0.001	1.445 (1.138-1.835)	0.003
T2a	1.718 (1.373-2.150)	<0.001	1.565 (1.243-1.969)	<0.001
Group				
Surgery	Reference		Reference	
Surgery+Chemotherapy	0.721 (0.615-0.844)	<0.001	0.748 (0.635-0.882)	<0.001
Stage of BC				
I	Reference		Reference	
II	1.142 (1.007-1.294)	0.039	1.226 (1.070-1.404)	0.003
III	1.335 (1.062-1.677)	0.013	1.456 (1.134-1.870)	0.003
Histology of BC				
IDC/NST	Reference		Reference	
Good Prognosis Tissue	0.897 (0.710-1.134)	0.365	0.885 (0.692-1.132)	0.330
ER				
Positive	Reference		Reference	
Negative	0.980 (0.858-1.120)	0.801	1.001 (0.804-1.248)	0.991
Borderline/Unknown	1.209 (0.984-1.485)	0.012	1.530 (0.916-2.555)	0.104
PR				
Positive	Reference		Reference	
Negative	0.984 (0.861-1.125)	0.768	0.999 (0.843-1.185)	0.995
Borderline/Unknown	1.216 (0.990-1.494)	0.071	0.804 (0.501-1.290)	0.366
HER2				
Positive	Reference		Reference	
Negative	1.306 (0.847-2.011)	0.227	1.164 (0.751-1.804)	0.497
Borderline/Unknown	1.751 (1.156-2.651)	0.008	1.435 (0.939-2.192)	0.095
Grade of BC				
Grade I	Reference		Reference	
Grade II	0.889 (0.768-1.03)	0.117	0.902 (0.775-1.050)	0.184
Grade III	0.943 (0.801-1.111)	0.485	0.963 (0.801-1.159)	0.694
Grade IV	1.897 (1.179-3.053)	0.008	1.629 (0.998-2.660)	0.010
Unknown	0.906 (0.713-1.151)	0.420	0.957 (0.748-1.224)	0.725
Chemotherapy of BC				
Yes	Reference		Reference	
No/Unknown	1.169 (1.028-1.329)	0.017	1.104 (0.946-1.290)	0.210
Radiation of BC				
Yes	Reference		Reference	
No/Unknown	1.186 (1.055-1.334)	0.004	1.093 (0.967-1.235)	0.153
Surgery of BC				
Yes	Reference		Reference	
No/Unknown	1.073 (0.664-1.732)	0.774	1.062 (0.651-1.733)	0.809

### 3.3 Survival analysis

**Figure 1:**
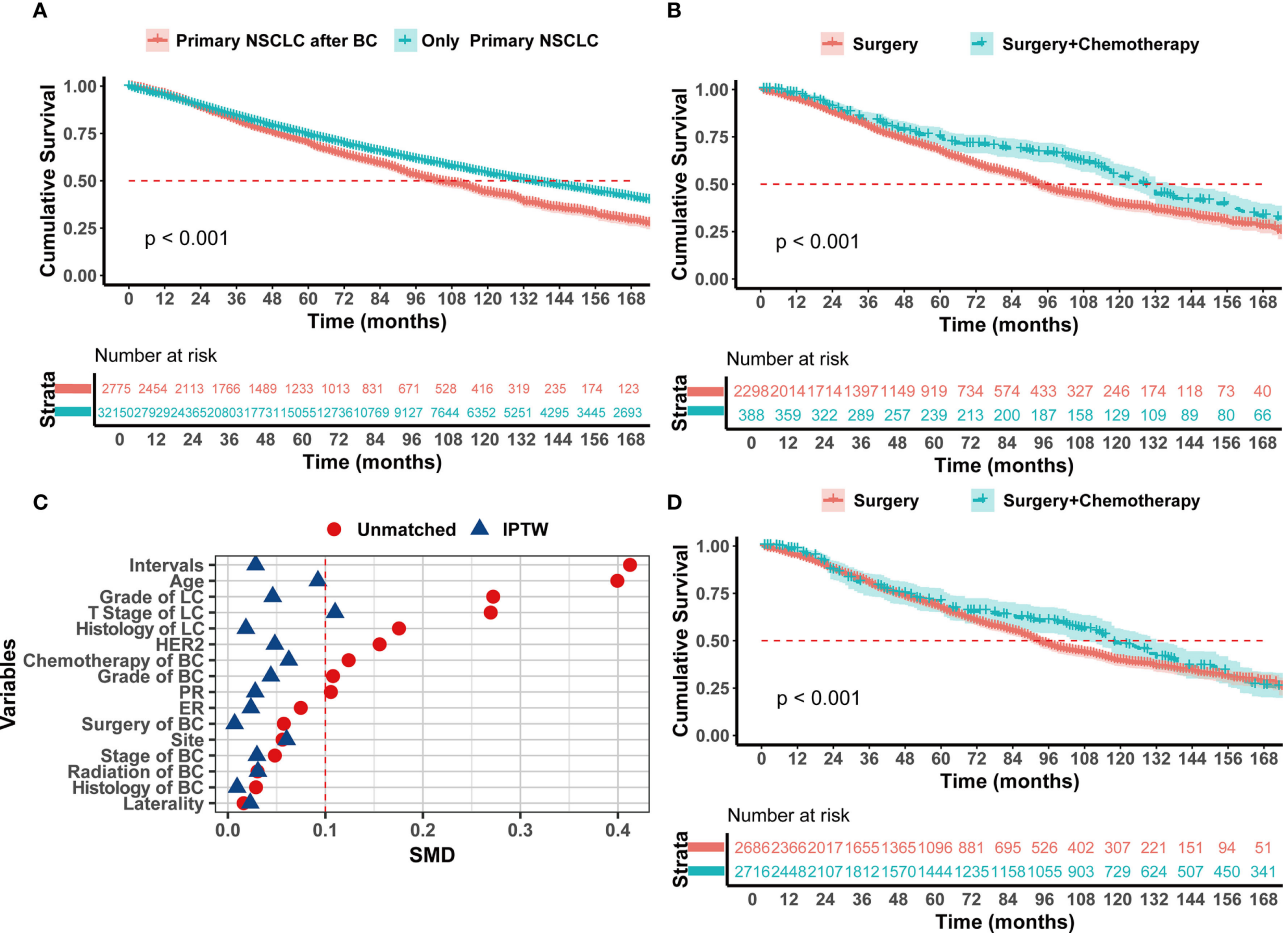
Kaplan–Meier analysis with log-rank testing of **(A)** Only Primary I NSCLC patients (n=32150; HR = 0.786; 95% CI, 0.740–0.834; P<0.001) versus Primary I NSCLC with prior BC patients (n=2775); **(B)** Primary I NSCLC with prior BC patients treated with Surgery + Chemotherapy (n=388; HR = 0.665; 95% CI, 0.562–0.787; P<0.001) versus Surgery (n=2298) **(C)** SMD before and after IPTW **(D)** Kaplan–Meier analysis with log-rank testing after IPTW patients treated with Surgery + Chemotherapy (n=2716; HR = 0.721; 95% CI, 0.615–0.844; P<0.001) versus Surgery (n=2686).

This study compared the survival of patients with secondary primary lung cancer to those with primary lung cancer. The results showed that the prognosis of patients with primary early-stage lung cancer was significantly better than that of patients with secondary primary early-stage lung cancer (HR = 0.786, 0.740-0.834, P < 0.001) (**Figure 1A**). Survival analysis using the Kaplan-Meier method demonstrated that the median survival time for the Surgery + Chemotherapy group and the Surgery group was129 months and 93 months, respectively (HR = 0.665, 0.562-0.787, p<0.001) (**Figure 1B**). To mitigate the impact of confounding factors on the outcomes and to ascertain the significance of radiotherapy and chemotherapy, an IPTW matching analysis was performed. The analysis indicated that all baseline characteristics were adequately matched, with standardized differences for variables other than T stage being less than 0.1 (**Figure 1C**). Within the matched analysis, the Surgery + Chemotherapy group continued to exhibit an improved prognosis, with a median survival time of 118 months, which was significantly longer than the 93 months observed in the Surgery group (HR = 0.721, 0.615-0.844, p<0.001) (**Figure 1D**).

### 3.4 Subgroup analysis

To determine which patients could benefit from postoperative chemotherapy, this study conducted a subgroup analysis based on age, interval between tumors, pathological type of lung cancer, lung cancer grade, lung cancer T stage, previous breast cancer stage, and history of breast cancer-related radiotherapy and chemotherapy. Breast Cancer Stage: For breast cancer stages I-II, the Surgery + Chemotherapy group had a significantly longer OS than the Surgery group (p < 0.05) (**Supplementary Figures S2A, B**). Despite no statistically significant difference in stage III breast cancer patients, the median survival time in the Surgery + Chemotherapy group (125 months) was markedly superior to that in the Surgery group (90 months) (**Supplementary Figure S2C**). Effect of Breast Cancer related radiotherapy and chemotherapy on lung cancer: Patients derived benefits from chemotherapy for lung cancer, irrespective of prior breast cancer-related radiotherapy (p < 0.05) (**Supplementary Figures S3A, B**). For patients who had not undergone breast cancer-related chemotherapy, significant survival benefits were observed when they received chemotherapy for lung cancer (126 months *vs* 90 months, HR = 0.696, 95% CI 0.573-0.846, p < 0.001) (**Supplementary Figure S3C**). In patients who had received chemotherapy for breast cancer, there was an observed extension in the medical survival time chemotherapy (130 months *vs* 106 months). However, this difference did not reach statistical significance (**Supplementary Figure S3D**). Lung Cancer T Stage: For T1a patients, the additional Chemotherapy did not prolong survival compared to Surgery (123 months *vs* 130 months, HR = 0.883, 0.493-1.580, p=0.67) (**Supplementary Figure S4A**). For T1b, the median survival time with Surgery + Chemotherapy was longer than with Surgery (132 months *vs* 97 months, p=0.005) (**Supplementary Figure S4B**), and while T1C patients also experienced an increase in median survival time chemotherapy (121 months *vs* 88 months), this increase was not statistically significant (**Supplementary Figure S4C**). As for T2a patients, the median survival time with Surgery + Chemotherapy was longer than with Surgery (118 months *vs* 83 months, p<0.001) (**Figures 3A**). Pathological Type of Lung Cancer: Chemotherapy benefits were noted in both adenocarcinoma and squamous cell carcinoma (p <0.05) (**Supplementary Figures S5A, B**). Lung Cancer Grade: The median survival time of Surgery + Chemotherapy was longer than Surgery in grades I-II (136 months *vs* 101 months, HR = 0.706, 0.568-0.878, p<0.001) (**Supplementary Figure S5C**). In patients with grades II-IV, the median survival time was significantly extended after chemotherapy (117 months *vs* 64 months, HR = 0.589, 95% CI 0.443-0.783, p < 0.001) (**Supplementary Figure S5D**). Age: The X-Tile method divided the cohort into two groups: age ≤ 70 years (n=1646) and age > 70 years(n=883). For patients aged ≤ 70 years, the median survival time of the Surgery + Chemotherapy group was significantly longer than the Surgery group alone (157 months *vs* 116 months, HR = 0.710, 95% CI 0.572-0.881, p = 0.002) (**Figure 3B**). In contrast, for patients aged > 70, there was no significant difference between the two groups (85 months *vs* 81 months, HR = 0.994, 95% CI 0.786-1.257, p = 0.95) (**Supplementary Figure S4D**). Interval Time: For patients with a tumor interval time ≤ 24 months, Surgery + Chemotherapy showed a better prognosis trend (132 months *vs* 97 months, HR = 0.616, 95% CI 0.487-0.780, p < 0.001) (**Figure 3C**). For patients with a tumor interval time > 24 months, Surgery + Chemotherapy did not extend postoperative survival time (117 months *vs* 90 months, HR = 0.877, 95% CI 0.707-1.088, p = 0.23) (**Figure 3D**).

**Figure 2:**
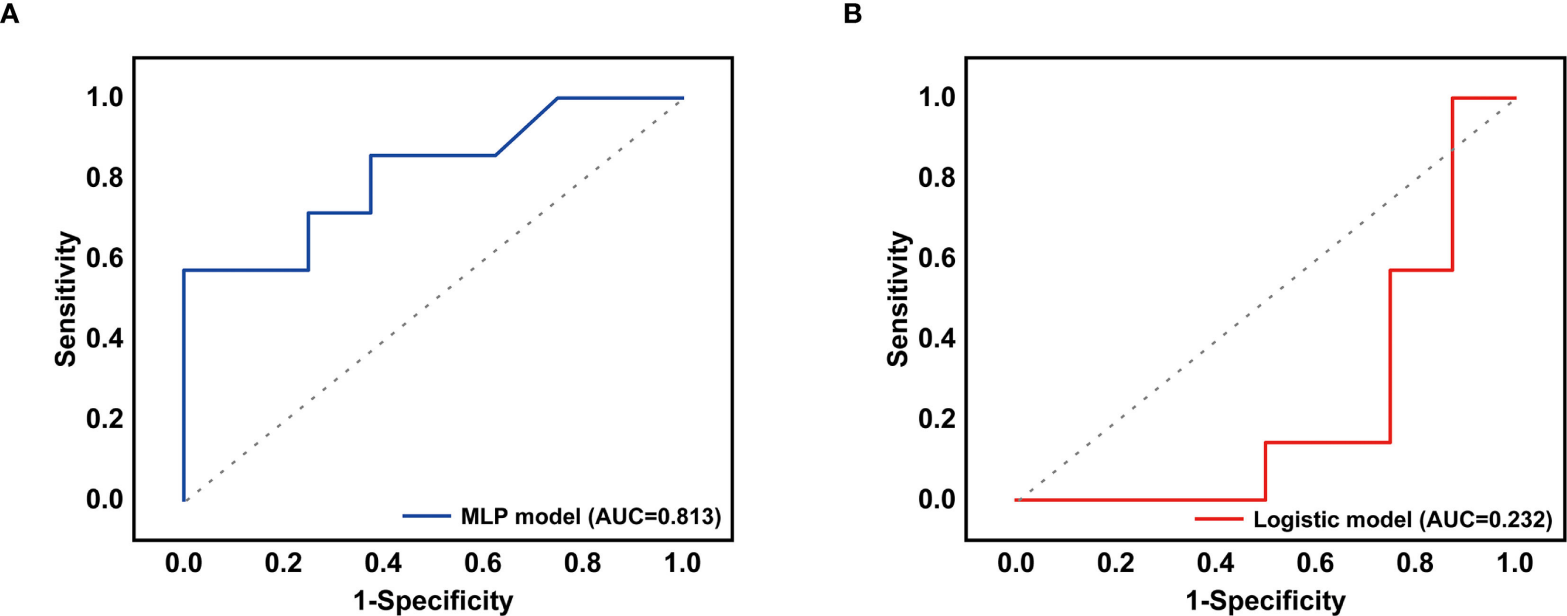
**(A)** The receiver operating characteristic curve for the MLP-lung model. **(B)** The receiver operating characteristic curve for the LOG-lung model.

**Figure 3:**
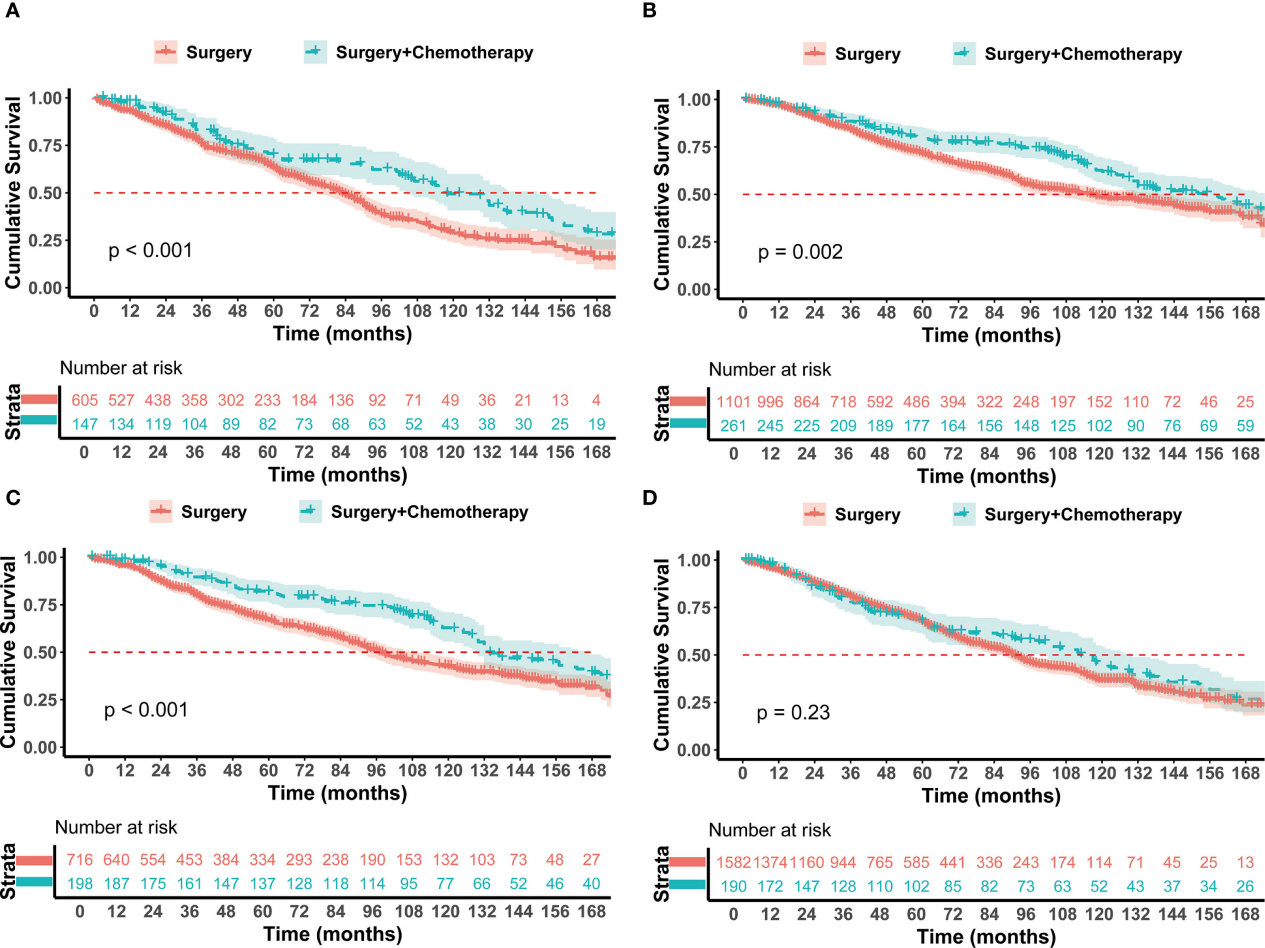
Kaplan–Meier analysis with log-rank testing of **(A)** T2a NSCLC patients treated with Surgery + Chemotherapy (n=147; HR = 0.625; 95% CI, 0.480–0.814; P<0.001) versus Surgery (n=605) **(B)** Age ≤ 70 yrs treated with Surgery + Chemotherapy (n=261; HR = 0.710; 95% CI, 0.572–0.881; P = 0.002) versus Surgery (n=1101) **(C)** Interval ≤ 24 months treated with Surgery + Chemotherapy (n=198; HR = 0.6169; 95% CI, 0.487–0.780; P<0.001) versus Surgery (n=716) **(D)** Interval > 24 months treated with Surgery + Chemotherapy (n=190; HR = 0.877; 95% CI, 0.707–1.088; P = 0.23) versus Surgery (n=1582).

### 3.5 Predictive and therapeutic decision-making models

**Figure 4:**
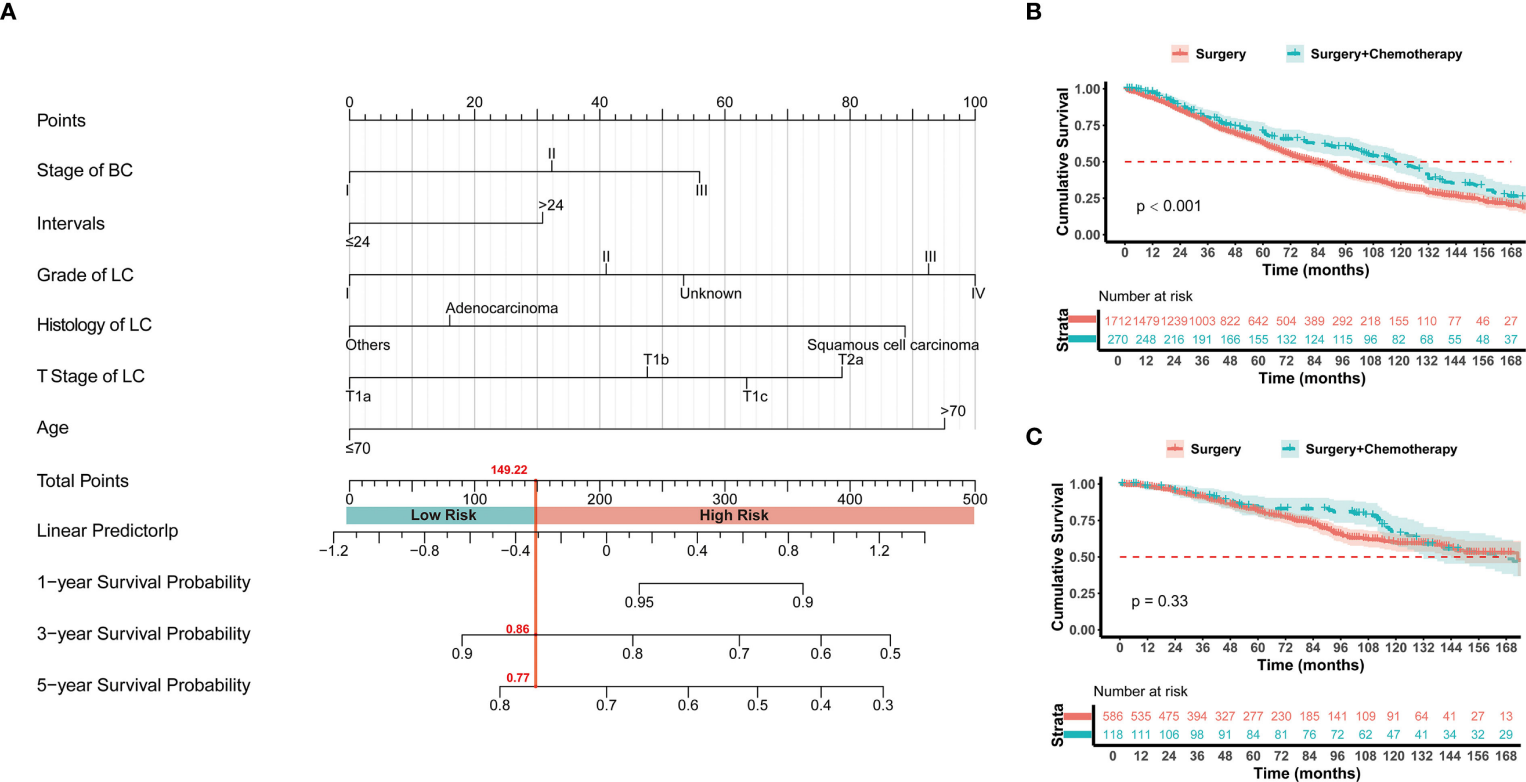
**(A)** The established nomogram model for predicting 1-, 3-, and 5-year survival in stage I NSCLC with prior BC patients. The patients were divided into high risk and low risk groups according to the score of 149.22. Kaplan–Meier analysis with log-rank testing of **(B)** high risk group treated with Surgery + Chemotherapy (n=270; HR = 0.721; 95% CI, 0.603–0.862; P<0.001) versus Surgery (n=1712); **(C)** low risk group treated with Surgery + Chemotherapy (n=118; HR = 0.843; 95% CI, 0.596–1.193; P = 0.33) versus Surgery (n=586).

Based on the hazard factors identified through COX model analysis, this study selected 6 non-treatment-related indicators: age, tumor interval time, histology of lung cancer, lung cancer grade, lung cancer T stage, and previous breast cancer stage, to construct the COX-lung therapeutic decision-making models. The model constructed a nomogram (**Figure 4A**) for predicting survival time with scoring rules for each indicator (**Supplementary Table S3**). After 1000-fold cross-validation, the model achieved a C-index of 0.629, and the calibration curve demonstrated good predictive accuracy for the overall survival rates at 1 year, 3 years, and 5 years (**Supplementary Figure S6**). A total of 2686 patients from the SEER database were used as the training set and 15 patients from the First Affiliated Hospital of Xi’an Jiaotong University served as the validation set. According to the scoring rules, patients were divided into high-risk and low-risk groups (risk score ≤ 149.22 for the low-risk group, > 149.22 for the high-risk group). Chemotherapy was recommended for the high-risk group, while it was not advised for the low-risk group. Kaplan-Meier survival analysis revealed that the median survival time for the high-risk group with additional chemotherapy was 118 months, which was significantly better than the 82 months for the Surgery group (HR = 0.721, 95% CI 0.603-0.862, p < 0.001), whereas there was no significant difference in median survival time between the chemotherapy and non-chemotherapy groups for the low-risk population (173 months *vs*. 166 months, HR = 0.843, 95% CI 0.596-1.193, p = 0.33) (**Figures 4B, C**). The study used a median survival time of 90 months for the non-chemotherapy group as the threshold for chemotherapy benefit. In the validation set, the COX-lung model had a positive predictive value of 75.00% and a negative predictive value of 57.72%, with the confusion matrix presented in **Supplementary Figure S7A**.

Furthermore, the 6 non-treatment-related indicators also were used to developed treatment decision-making models including the MLP-lung and the LOG-lung via machine learning. The MLP-lung model had an AUC of 0.813 in the validation set (**Figure 2A**), and the confusion matrix is depicted in **Supplementary Figure S7B**, showing a positive predictive value of 100.00% and a negative predictive value of 57.72%. The LOG-lung model, utilizing the logistic regression algorithm, yielded an AUC of 0.2322 (**Figure 2B**), with a positive predictive value of 50.00% and a negative predictive value of 14.29% (**Supplementary Figure S7C**). The significance scores of the training features for the MLP-lung model revealed that age, interval time between tumors, and lung cancer T stage were pivotal in predicting the benefits of chemotherapy (**Supplementary Figure S8**). SHAP analysis interpreted the MLP-Lung model’s predictions, wherein the swarm plot (**Supplementary Figure S9A**) visualizes feature impact directionality and magnitude using a blue-to-red spectrum denoting low-to-high feature values, while the summary plot (**Supplementary Figure S9B**) reveals age ≤70 years as the dominant contributor, followed by >24-month intervals and age >70 years, consistent with clinical priorities.

## 4 Discussion

Currently, the long-term OS for completely resected stage I NSCLC patients remain suboptimal. Although the evidence supporting adjuvant chemotherapy for stage I NSCLC is still insufficient, incorporating systematic treatment may be necessary to improve patient outcomes, particularly for those with high-risk factors (9, 10). A propensity score-matched analysis in the United States indicated that adjuvant chemotherapy significantly improved the 5-year OS by 8%; furthermore, in the cohort of patients with stage IB NSCLC, those with tumor sizes ranging from 3.1 to 3.9 cm also displayed extended survival with adjuvant chemotherapy (11). In the treatment of recurrent tumors, some clinicians may prefer conservative treatment regimens, while others may follow the NCCN. However, considering the heterogeneity of this population and their relatively poor prognoses, the necessity of exploring alternative treatment strategies, such as adjuvant chemotherapy or targeted therapies, warrants further investigation.

This study’s survival analysis revealed that stage I SPLC patients with a history of prior breast cancer exhibited poorer prognoses, experiencing significantly lower survival rates compared to stage I first primary lung cancer. These findings are consistent with previous research (12, 13). The cohort of patients with early-stage lung cancer who have a history of breast cancer displays heterogeneity, leading to variability in prognoses and treatment responses among different individuals (14). According to the TNM staging system, the reported 5-year OS rate for patients with pathological stage I NSCLC ranges from 65.3% to 74.9%, depending on various studies (15). Thus, traditional treatment strategies may not be applicable. Due to the heterogeneity among these patients, it is essential to explore the risk factors influencing the survival of individuals with stage I SPLC patients, as well as to identify the most effective treatment strategies.

This study analyzed the SEER database and found that the pathological characteristics of previous breast cancer, HER2 expression status, hormone receptor status, and pathological grade did not significantly affect the mortality risk of patients with recurrent stage I NSCLC. These findings suggest that the prognosis of such patients may not be influenced by the tumor classification of breast cancer or the expression of hormone receptors. However, the stage of breast cancer remains a critical risk factor for the prognosis of patients with second primary lung cancer. This result is understandable, given that there are significant differences in prognosis among breast cancer patients at various stages (16).

For the analysis of treatment methods, 1/8 patients received chemotherapy, suggesting that in the real world, doctors and patients choose adjuvant chemotherapy, and the results suggest that some people have benefited from adjuvant chemotherapy. This finding suggests that some individuals may have benefited from this treatment. To minimize the influence of other risk factors, this study employed IPTW. Following baseline matching, the benefits of chemotherapy remained evident, potentially offering new insights for the treatment of these patients.

Furthermore, subgroup analysis results indicate that the population benefiting from chemotherapy possesses specific clinical characteristics, suggesting that these patients require further stratified analysis to identify differences in prognosis and treatment response. Regarding the staging of breast cancer, patients with non-metastatic breast cancer may still derive benefits from lung cancer-related adjuvant chemotherapy; however, the statistical difference in chemotherapy benefits for stage III breast cancer patients is not yet significant, potentially due to a smaller sample size. As breast cancer staging progresses, the median survival time of patients benefiting from additional chemotherapy increases from 28 months to 35 months compared to those receiving surgery alone. Patients with more advanced breast cancer stages exhibit a higher risk of lung cancer, therefore likely accruing greater benefits from chemotherapy. Patients previously treated with breast cancer-related chemotherapy do not demonstrate significant benefits from lung cancer-related chemotherapy (p=0.089), which may be attributed to chemotherapy resistance resulting from breast cancer treatments. Chemotherapy may influence the host’s overall condition through various mechanisms (17). Additionally, there is some overlap between lung cancer-related and breast cancer-related chemotherapeutic agents; for example, the combination of paclitaxel and platinum-based chemotherapy regimens is utilized in the treatment of both malignancies (18, 19), which may diminish the efficacy of lung cancer-related chemotherapy.

In terms of lung cancer characteristics, patients with T2a tumors or moderate differentiation may benefit from chemotherapy due to their higher risk and more aggressive disease. Patients aged below 70 may experience benefits from chemotherapy, whereas those over 70 do not show similar advantages. This study posits that this disparity could relate to life expectancy and overall physical condition. The interval between disease occurrences is another important factor to consider. Shoji et al. (20) reported on 14 patients with concurrent breast and lung cancer, all of whom had lung cancer diagnosed within three years of undergoing breast cancer surgery, with a five-year survival rate of 22.2% following the diagnosis of the second primary lung cancer. This finding suggests that lung cancer recurrences occurring in the short term may be more closely associated with breast cancer and carry a higher risk. However, patients with stage I lung cancer recurring more than two years after breast cancer diagnosis do not benefit significantly from chemotherapy regarding prognosis. This indicates that patients with shorter interval times derive greater value from chemotherapy, which may be due to the similar growth characteristics and genetic backgrounds shared between the recurrent tumors and breast cancer. Conversely, patients with longer interval times are more likely to exhibit independently originating lung cancer, making the benefits of chemotherapy align more closely with the NCCN guidelines for primary lung cancer.

Given the heterogeneity of such patients and the variability in chemotherapy benefits, this study constructed a survival prediction model and three treatment decision models to assist clinicians in identifying high-risk patient groups that may benefit from chemotherapy. Based on the results of COX regression analysis, six non-treatment-related characteristics were selected for survival prediction and treatment decision-making. First, a nomogram predicting survival was created using these six characteristics, and its predictive accuracy was evaluated through cross-validation and calibration curves. Subsequently, three treatment decision models were developed: the COX-lung model, the MLP-lung model, and the LOG-lung model, each designed to predict the population that would benefit from chemotherapy. All three models categorized patients into high-risk and low-risk groups; however, due to the rarity of such patients, this study collected data from only 15 patients with stage I lung cancer following breast cancer for validation. The COX-lung model is based on a scoring system derived from the COX proportional hazards model nomogram (**Figure 4A**), allowing clinicians to group patients conveniently for clinical application. The LOG-lung model, built on a classical logistic regression algorithm, exhibited relatively low accuracy. The MLP-lung model, developed using the MLP algorithm, achieved high predictive accuracy, potentially due to its ability to learn complex nonlinear relationships and interactions (21). This study analyzed the importance of features in the MLP-lung model, identifying age, interval time, and lung cancer T staging as key characteristics for predicting populations that would benefit from chemotherapy.

With the increasing number of long-term survivors of early breast cancer, there is an urgent need to investigate the biological mechanisms underlying tumor behavior in this population. For instance, examining the correlation between lung and breast cancer development is vital. Patients with secondary primary stage I NSCLC and a history of non-metastatic breast cancer represent a unique subgroup. Breast and lung cancers may share comparable genetic foundations and growth characteristics, potentially elevating the risk of lung cancer in breast cancer survivors. Dysregulation of specific genes, such as TP53 and EGFR, has been implicated in both breast and lung cancers (22–24), indicating that these genes may be crucial in the development of multiple primary cancers. Mutations in the BRCA1/2 genes are linked not only to the onset of breast cancer but also have a strong correlation with the development of lung cancer (25, 26). Enhanced transcriptional activity of AGER and RAGE may contribute to an increased risk of breast and lung cancers (27, 28). These findings, to a certain degree, illustrate the shared genetic predisposition to both breast and lung cancers due to mutations. Additionally, breast cancer therapies could result in immunosuppression, diminishing the body’s tumor defense mechanisms and potentially raising the risk of subsequent primary tumors. Radiation therapy’s direct harm to lung tissue might elevate the risk of carcinogenesis in the lungs (29, 30),although the risk of complications from radiotherapy may decrease with advancements in technology (31). Overall, a history of breast cancer is significantly associated with the subsequent development of lung cancer. Several researchers have examined the risk factors for the development of SPLC in breast cancer patients, including age, time interval, smoking, ER status, HER2 status, and PR status (32–35).

This study has several limitations that warrant consideration. First, our institutional cohort was relatively small (n = 15) due to the rarity of this clinical scenario, which limits statistical power and prevents robust comparisons with the SEER population. Although we applied consistent inclusion criteria to both datasets to minimize bias, differences in data resolution and the potential for unmeasured confounders between institutional and registry sources remain. Second, the absence of genomic information—particularly regarding common driver mutations such as EGFR or ALK—is a major limitation. This is primarily due to the lack of molecular annotations in the SEER database and the small number of genotyped cases available at our institution. In current clinical practice, molecular alterations play a central role in treatment selection and prognostic assessment for NSCLC. Without this information, the model lacks granularity and cannot fully reflect how real-world decisions are made in personalized oncology.

This issue is especially relevant given the growing use of targeted therapies in early-stage NSCLC. Recent clinical trials such as ADAURA and ALINA have demonstrated the benefit of adjuvant targeted treatments in patients with actionable mutations (36, 37). However, stage I patients with a prior cancer history have been largely excluded from these trials. In routine care, managing these cases—particularly when considering a potential SPLC—often requires careful, individualized discussion within a multidisciplinary tumor board (38, 39). This again highlights the importance of incorporating molecular data into predictive models to ensure alignment with current clinical reasoning and practice.

To address these issues, we are initiating a prospective, multi-center study that will include genomic profiling. This will allow us to evaluate model performance in a more diverse and molecularly defined patient population. Additionally, we plan to analyze TCGA datasets to investigate potential molecular links between primary breast cancers and subsequent lung malignancies. This may provide deeper biological insights and improve the interpretability of the model, particularly in patients with a history of prior cancer. Our study found that chemotherapy can significantly improve outcomes for breast cancer patients with secondary early-stage lung cancer, a previously underexplored area. We utilized a deep learning model called MLP-lung, which effectively uncovers complex nonlinear relationships among patient characteristics and treatment outcomes. This innovative approach not only improved predictive accuracy but also lays a novel framework for individualized chemotherapy decision-making. However, the existing research in this field has faced limitations, including small sample sizes, restricted generalizability, and inadequate integration of clinical variables with advanced predictive models. Our study addresses these gaps by incorporating data from the SEER database and independent validation samples, enhancing broader applicability across diverse patient populations. The integration of the MLP-lung model marks a paradigm shift in assessing treatment benefits. While traditional statistical methods often struggle to capture intricate feature interactions, our deep learning model offers superior flexibility and precision. This advancement sets the foundation for future studies aimed at optimizing treatment approaches in various multi-cancer scenarios. Importantly, our findings underscore the necessity for personalized chemotherapy strategies in this patient population, moving away from one-size-fits-all approaches. The predictive capabilities of the MLP-lung model inform clinical decision-making and hold potential for integration into multidisciplinary treatment planning systems. In summary, while our research introduces a novel methodological approach and provides critical insights, we recognize its limitations. The relatively small external validation cohort (15 samples) highlights the necessity for further validation using larger, multi-institutional datasets. Future investigations should also explore the molecular mechanisms underlying the observed chemotherapy benefits, enriching the biological understanding that complements our predictive framework. By integrating robust data, advanced predictive analytics, and focusing on a clinically significant research area, this work not only fills an important void but also establishes groundwork for more effective, patient-centered treatment strategies in the future.

## 5 Conclusion

SPLC patients have poorer prognosis; however, adjuvant chemotherapy can improve outcomes for some of these patients. The COX-lung model and the MLP-lung model can identify patients who may benefit from chemotherapy, thereby providing important reference points for clinicians in developing individualized treatment plans. Future research should focus on further validating the reliability and effectiveness of these models, as well as exploring alternative prognostic prediction methods to enhance survival rates for patients with a history of breast cancer who subsequently develop early lung cancer.

## Data Availability

The raw data supporting the conclusions of this article will be made available by the authors, without undue reservation.
