# Supplementary material for: Does chemotherapy improve survival outcomes in breast cancer survivors with secondary primary stage I non-small cell lung cancer? A real-world analysis using machine learning models
Source: Front Oncol. 2025 Sep 12;15:1646580. doi: 10.3389/fonc.2025.1646580 (PMC12463641; doi:10.3389/fonc.2025.1646580)
Supplement: Supplementary file 1 [file DataSheet1.pdf]

Supplementary Tables

Table S1: I Stage NSCLC Patients Baseline Characteristics in SEER Database.

Characteristics	Primary NSCLC after BC n =2775(%)	Only Primary NSCLC n =32150(%)	c^2	P
**Age at diagnosis**				
≤70	1406 (50.67)	19500 (60.65)	106.028	<0.001
>70	1369 (49.33)	12650 (39.35)		
**Histology of LC**				
Adenocarcinoma	1792 (64.58)	20481 (63.70)	12.556	0.002
Squamous cell carcinoma	485 (17.48)	5092 (15.84)		
Others	498 (17.95)	6577 (20.46)		
**Grade of LC**				
Grade I	598 (21.55)	7394 (23.00)	34.706	<0.001
Grade II	1219 (43.93)	12808 (39.84)		
Grade III	565 (20.36)	6716 (20.89)		
Grade IV	12 (0.43)	466 (1.45)		
Unknown	381 (13.73)	4766 (14.82)		
**Laterality**				
Left - origin of primary	1162 (41.87)	12824 (39.89)	5.576	0.032
Right - origin of primary	1613 (58.13)	19309 (60.06)		
Unknown	0 (0.00)	17 (0.05)		
**Primary Site**				
Upper lobe	1667 (60.07)	18341 (57.05)	21.544	<0.001
Middle lobe	144 (5.19)	2203 (6.85)		
Lower lobe	924 (33.30)	10903 (33.91)		

Others	40 (1.44)	703 (2.19)		
**T Stage of LC**				
T1a	361 (13.01)	5113 (15.90)	67.540	<0.001
T1b	1073 (38.67)	10181 (31.67)		
T1c	574 (20.68)	7858 (24.44)		
T2a	767 (27.64)	8998 (27.99)		

Table S2: I Stage NSCLC Patients Baseline Characteristics in our Database.

Characteristics	Primary NSCLC after BC n =15(%)
**Age at diagnosis**	
≤70	9 (60.00)
>70	6 (40.00)
**Intervals**	
≤24	7 (46.67)
>24	8 (53.33)
**Histology of LC**	
Adenocarcinoma	10 (66.67)
Squamous cell carcinoma	3 (20.00)
Others	2 (13.33)
**Grade of LC**	
Grade I	2 (13.33)
Grade II	8 (53.33)
Grade III	4 (26.67)
Grade IV	1 (6.67)
**Laterality**	
Left - origin of primary	5 (33.33)
Right - origin of primary	10 (66.67)
**Primary Site**	
Upper lobe	12 (80.00)
Lower lobe	3 (20.00)
**T Stage of LC**	
T1a	1 (6.67)

T1b	6 (40.00)
T1c	2 (13.33)
T2a	6 (40.00)
**Stage of BC**	
I	8 (53.33)
II	6 (40.00)
III	1 (6.67)
**Histology of BC**	
IDC/NST	3 (20.00)
Good Prognosis Tissue	12 (80.00)
**ER**	
Positive	14 (93.33)
Negative	1 (6.67)
**PR**	
Positive	13 (86.67)
Negative	2 (13.33)
**HER2**	
Positive	6 (40.00)
Negative	9 (60.00)
**Grade of BC**	
Grade I	1 (6.67)
Grade II	10 (66.67)
Grade III	4 (26.67)
**Chemotherapy of BC**	
Yes	12 (80.00)

No	3 (20.00)
**Radiation of BC**	
Yes	8 (53.33)
No	7 (46.67)
**Surgery of BC**	
Yes	15 (100.00)
No	0 (0.00)

Table S3: Characteristics Scores.

Characteristics	Scores
Stage of BC I	0.00
Stage of BC II	32.33
Stage of BC III	55.95
Intervals ≤ 24	0.00
Intervals > 24	30.86
Grade I	0.00
Grade II	41.03
Grade III	92.53
Grade IV	100.00
Grade unknown	53.41
Adenocarcinoma	16.02
Squamous cell carcinoma	88.80
Others	0.00
T1a	0.00
T1b	47.60
T1c	63.48
T2a	78.71
Age ≤ 70	0.00
Age > 70	95.11
