# Supplementary material for: Does chemotherapy improve survival outcomes in breast cancer survivors with secondary primary stage I non-small cell lung cancer? A real-world analysis using machine learning models
Source: Front Oncol. 2025 Sep 12;15:1646580. doi: 10.3389/fonc.2025.1646580 (PMC12463641; doi:10.3389/fonc.2025.1646580)
Supplement: Supplementary file 2 [file DataSheet2.pdf]

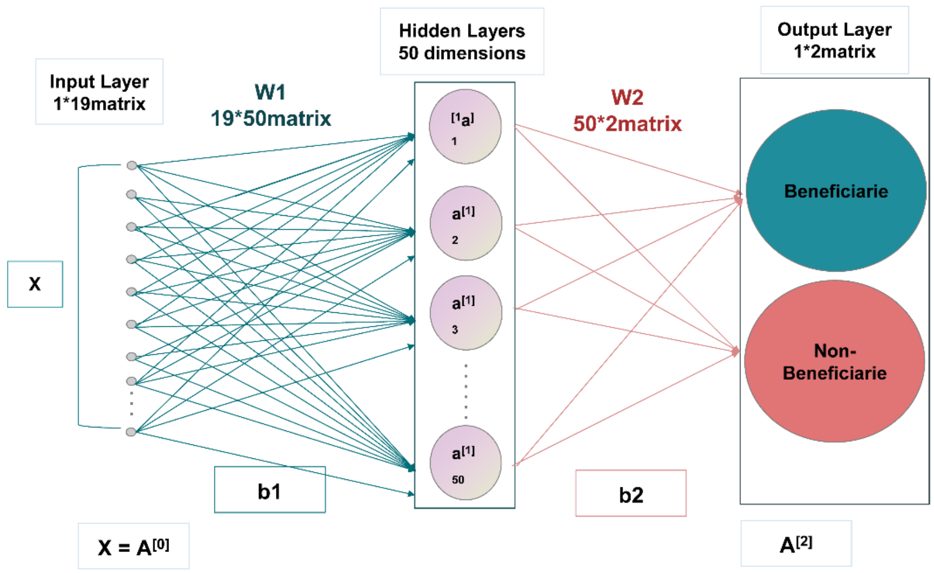

FigureS1. The MLP for patients with stage I NSCLC: The input layer represents the clinical features input into the deep learning model. The hidden layer is the deep learning neural network, where each node represents a neuron for calculation. The output layer is used to classify whether chemotherapy is suitable after the calculation by the neural network.

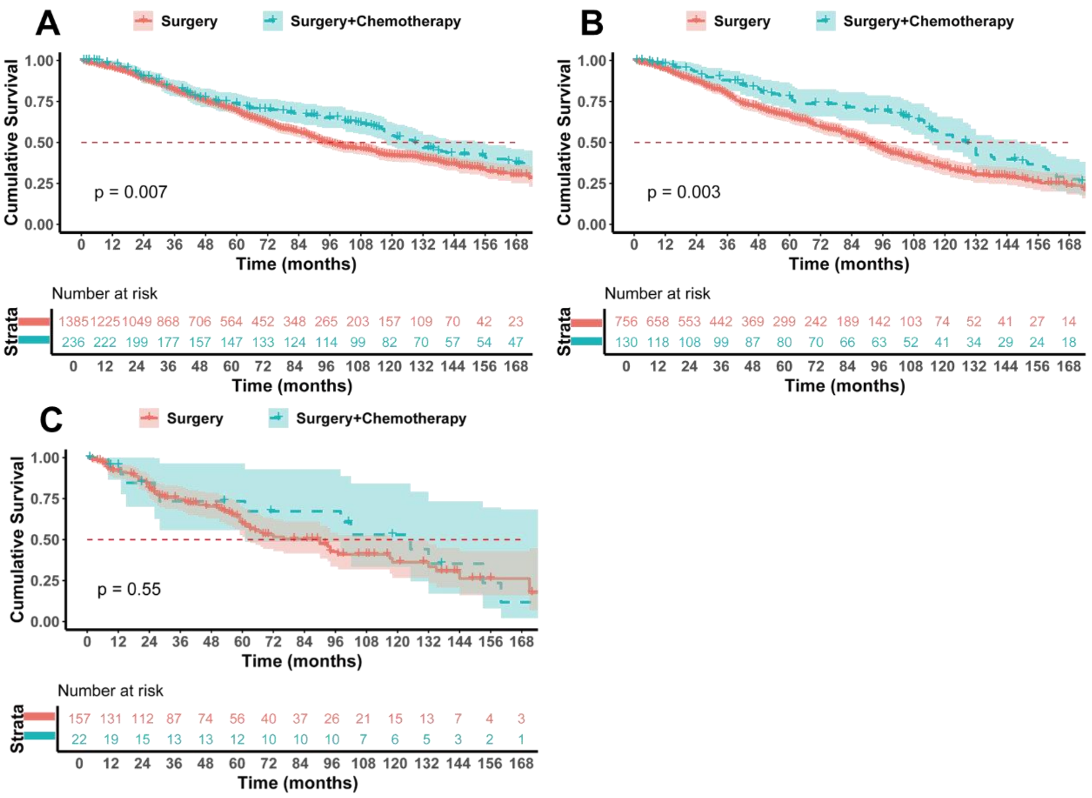

FigureS2. Kaplan–Meier analysis with log-rank testing of Primary I NSCLC with prior (A) Kaplan–Meier analysis after PSM patients treated with Surgery + Chemotherapy (n=387; HR=0.701; 95% CI,0.549-0.895; p=0.004) versus Surgery (n=387) (B) Stage I BC patients treated with Surgery + Chemotherapy (n=236; HR=0.753; 95% CI, 0.613–0.926; P=0.007) versus Surgery (n=1385) (C)

Stage II BC patients treated with Surgery + Chemotherapy (n=130; HR=0.662; 95% CI, 0.505–0.867; P<0.001) versus Surgery (n=756) (D) Stage III BC patients treated with Surgery + Chemotherapy (n=22; HR=0.828; 95% CI, 0.445–1.543; P<0.001) versus Surgery (n=157)

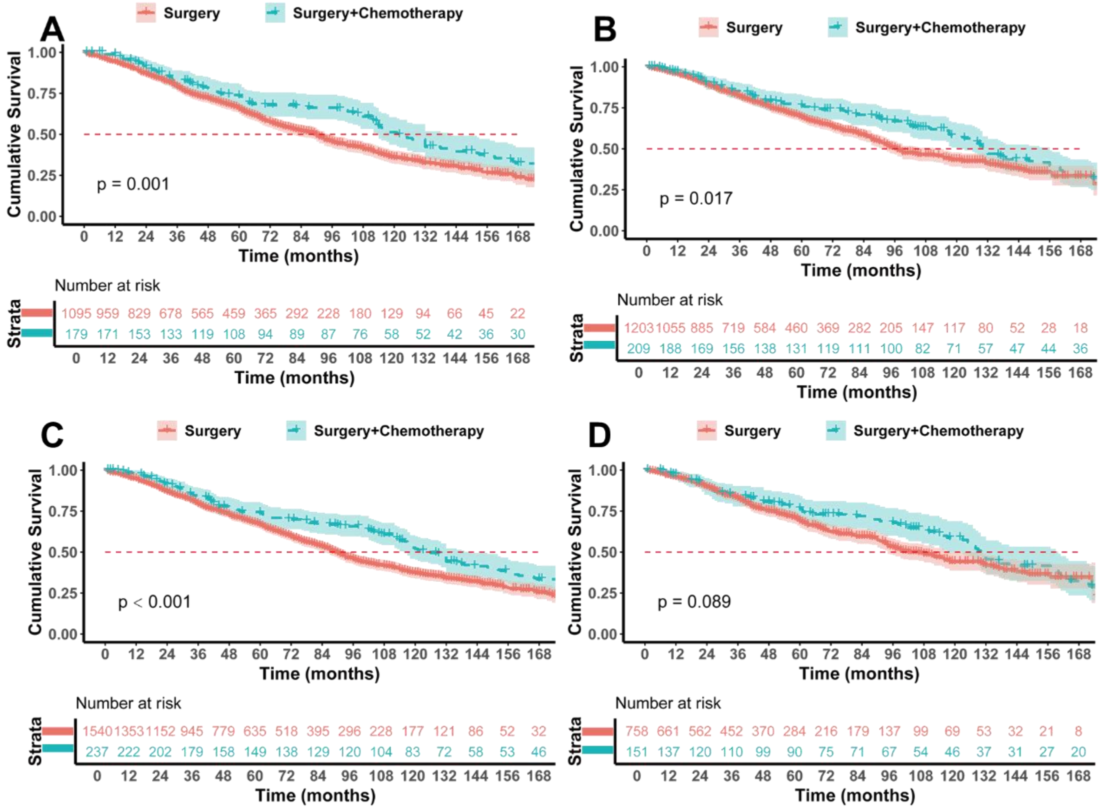

FigureS3. Kaplan–Meier analysis with log-rank testing of Primary I NSCLC with (A) non-radiotherapy for BC patients treated with Surgery + Chemotherapy (n=179; HR=0.693; 95% CI, 0.553–0.868; P<0.001) versus Surgery (n=1095) (B) radiotherapy for BC patients treated with Surgery + Chemotherapy (n=209; HR=0.761; 95% CI, 0.608–0.952; P=0.017) versus Surgery (n=1203) (C) non-chemotherapy for BC patients treated with Surgery + Chemotherapy (n=237; HR=0.696; 95% CI, 0.573–0.846; P <0.001) versus Surgery (n=1540) (D) chemotherapy for BC patients treated with Surgery + Chemotherapy (n=151; HR=0.789; 95% CI, 0.600–1.037; P=0.089) versus Surgery (n=758)

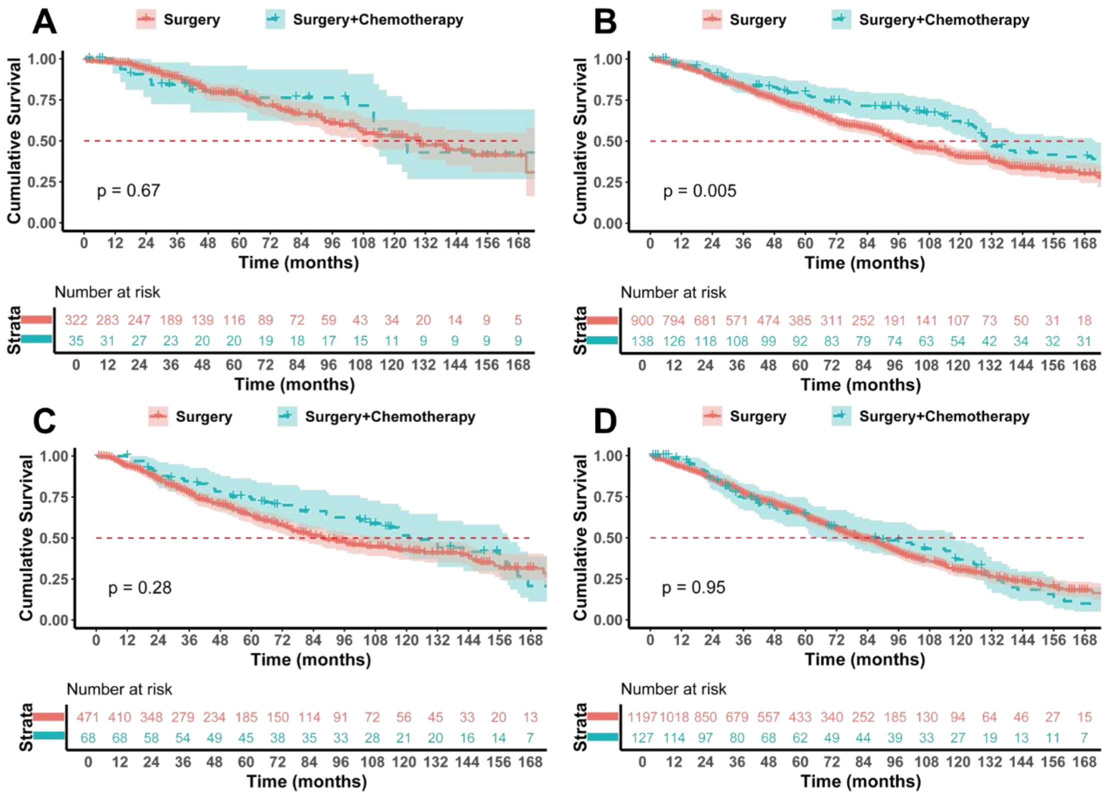

FigureS4. Kaplan–Meier analysis with log-rank testing of (A) T1a NSCLC patients treated with Surgery + Chemotherapy (n=35; HR=0.883; 95% CI, 0.494–1.580; P=0.67) versus Surgery (n=322) (B) T1b NSCLC patients treated with Surgery + Chemotherapy (n=138; HR=0.683; 95% CI, 0.523–0.892; P=0.005) versus Surgery (n=900) (C) T1c NSCLC patients treated with Surgery + Chemotherapy (n=88; HR=0.829; 95% CI, 0.588–1.167; P=0.28) versus Surgery (n=471) (D) Age > 70 yrs treated with Surgery + Chemotherapy (n=127; HR=0.994; 95% CI, 0.786–1.257; P=0.95) versus Surgery (n=1197)

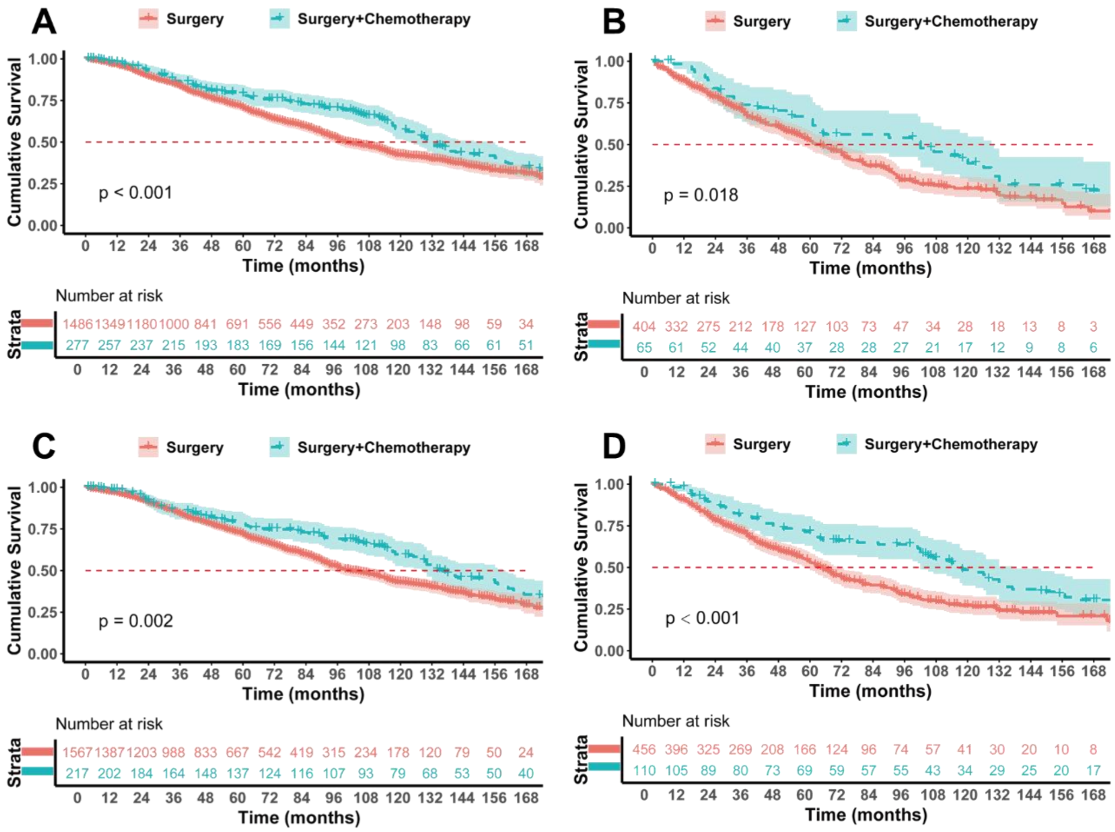

FigureS5. Kaplan–Meier analysis with log-rank testing of Primary I NSCLC (A) adenocarcinoma patients treated with Surgery + Chemotherapy (n=277; HR=0.721; 95% CI, 0.596–0.872; P<0.001) versus Surgery (n=1486) (B) squamous cell carcinoma patients treated with Surgery + Chemotherapy (n=65; HR=0.665; 95% CI, 0.473–0.935; P=0.018) versus Surgery (n=404) (C) Grade I-II patients treated with Surgery + Chemotherapy (n=237; HR=0.706; 95% CI, 0.568–0.877; P =0.002) versus Surgery (n=1567) (D) Grade III-IV patients treated with Surgery + Chemotherapy (n=110; HR=0.589; 95% CI, 0.444–0.783; P<0.001) versus Surgery (n=456)

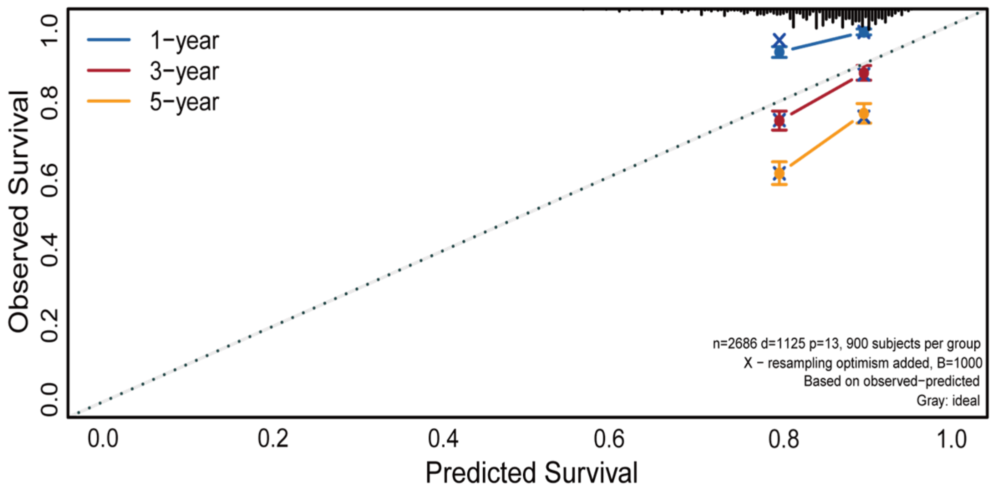

FigureS6. Calibration curve of the nomograms. a: 1-, 3-, and 5-year survival nomogram calibration curves. The results showed well agreement between prediction and observation in the probability.

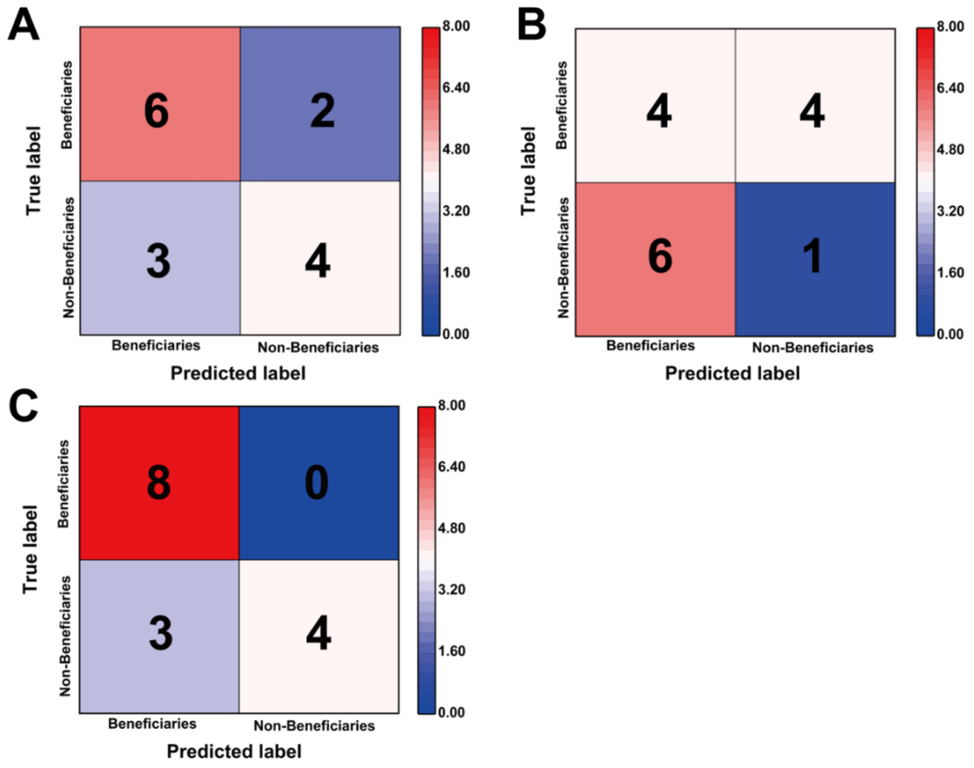

FigureS7. (A) The confusion matrix of the COX-lung model is depicted based on the outcomes of 15 patients. (B) The confusion matrix of the MLP-lung model is depicted based on the outcomes of 15 patients. (C) The confusion matrix of the LOG-lung model is depicted based on the outcomes of 15 patients.

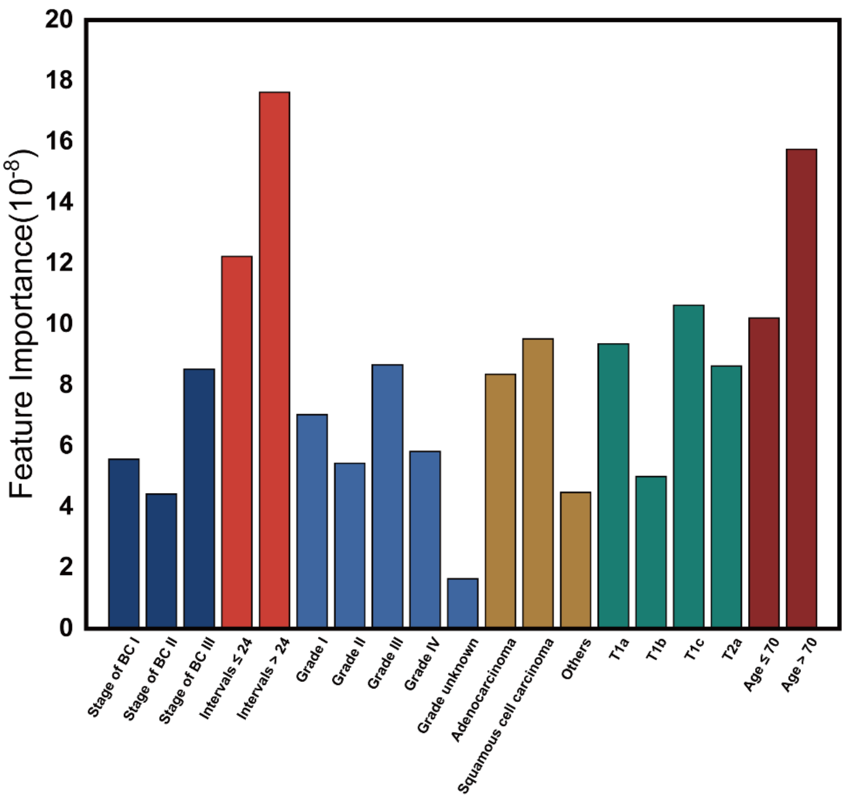

FigureS8. The analysis of feature importance based on the MLP-lung model.

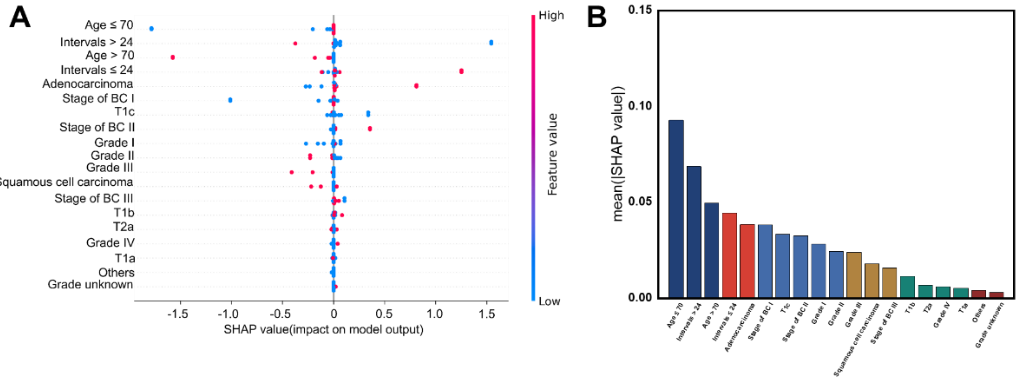

FigureS9. SHAP Value analysis for MLP-lung model (A) SHAP swarm plot. (B) The importance ranking of the 6 variables according to the mean (|SHAP value|).
